# Dependency of planned dose perturbation (PDP) on the spatial resolution of MapCHECK 2 detectors

**DOI:** 10.1120/jacmp.v15i1.4457

**Published:** 2014-01-06

**Authors:** Vance P. Keeling, Salahuddin Ahmad, Ozer Algan, Hosang Jin

**Affiliations:** ^1^ Department of Radiation Oncology University of Oklahoma Health Sciences Center Oklahoma City OK USA

**Keywords:** planar IMRT quality assurance, MapCHECK, 3DVH, planned dose perturbation, small‐field IMRT QA

## Abstract

The purpose of this study is to determine the dependency of the planned dose perturbation (PDP) algorithm (used in Sun Nuclear 3DVH software) on spatial resolution of the MapCHECK 2 detectors. In this study, ten brain (small target), ten brain (large target), ten prostate, and ten head‐and‐neck (H&N) cases were retrospectively selected for QA measurement. IMRT validation plans were delivered using the field‐by‐field technique with the MapCHECK 2 device. The measurements were performed using standard detector density (standard resolution; SR) and a doubled detector density (high resolution; HR) by merging regular with shifted measurements. SR and HR measurements were fed into the 3DVH software and ROI (region of interest), planning target volume (PTV), and organ at risk (OAR)) dose statistics ($D95,D_{\text{mean}}$. and $D_{\text{max}}$) were determined for each. Differences of the dose statistics normalized to prescription dose for ROIs between original planning and PDP‐perturbed planning were calculated for $\text{SR}\;(\Delta D_{\text{SR}})$ and $\text{HR}\;(\Delta D_{\text{HR}})$, and difference between $\Delta D_{\text{SR}}$ and $\Delta D_{\text{HR}}\;(\Delta D_{\text{SR}-\text{HR}}=\Delta D_{\text{SR}}-{\text{DLD}}_{\text{HR}})$ was also calculated. In addition, 2D and 3D γ passing rates (GPRs) were determined for both resolutions, and a correlation between GPRs and $\Delta D_{\text{SR}}$ or $\Delta D_{\text{HR}}$ for PTV dose metrics was determined. No considerably high mean differences between $\Delta D_{\text{SR}}$ and $\Delta D_{\text{HR}}$ were found for almost all ROIs and plans (<2%); however, $\left|\Delta D_{\text{SR}}|,|\Delta D_{\text{HR}}\right|$, and $\left|\Delta D_{\text{SR}-\text{HR}}\right|$ for PTV were found to significantly increase as the PTV size decreased (e.g., PTV size<5cc). And statistically significant differences between SR and HR were observed for OARs proximal to targets in large brain target and H&N cases. As plan modulation represented by fractional MU/prescription dose (MU/cGy) became more complex, the 2D/3D GPRs tended to decrease; however, the modulation complexity did not make any noticeable distinctions in the DVH statistics of PTV between SR and HR, excluding the small brain cases whose PTVs were extremely small (PTV=11.0±10.1cc). Moderate to strong negative correlations (−1<r<−0.3) between GPRs and PTV dose metrics indicated that small clinical errors for PTV occur at the higher GPRs. In conclusion, doubling the detector density of the MapCHECK 2 device is recommended for small targets (i.e., PTV<5cc) and multiple targets with complex geometry with minimum setup error in the DVH‐based plan evaluation.

PACS numbers: 87.55.dk, 87.55.kd, 87.55.km, 87.55.Qr, 87.56.Fc

## I. INTRODUCTION

Due to the complexity and uniqueness for intensity‐modulated radiation therapy (IMRT) plans, each plan must be verified through quality assurance (QA) tests. The most common way of performing IMRT QA is the quantitative comparison of measured dose distributions on a phantom with dose distributions generated by the treatment planning system (TPS) for an analogous setup,^(1)^ accomplished through the use of the γ test.^(2)^ The criteria used by a number of institutions for γ test are percent dose difference of 3% and distance‐to‐agreement (DTA) of 3 mm.^3^, ^4^, ^5^ A study was performed to determine accepted tolerance levels based on statistical analysis of numerous IMRT QA passing rates from different institutions.^(4)^ However, these studies were primarily based on what IMRT QA passing rates are achievable, and not based on what is clinically acceptable. Other studies have shown that planar IMRT QAs using γ passing rates (GPRs) are not good indicators of dose errors in patients.^6^, ^7^, ^8^, ^9^, ^10^, ^11^, ^12^

The main disadvantage of the 2D planar IMRT QA is the fact that the dose validation is accomplished on the phantom geometry and not on actual patient geometry. A planned dose perturbation (PDP) algorithm implemented in the Sun Nuclear Corporation (SNC; Melbourne, FL) 3DVH software is proposed to overcome this intrinsic drawback of the conventional IMRT QA technique.^(12)^ The PDP algorithm uses errors determined from the comparison of calculated distributions by TPS against measured distributions by a diode array detector (SNC MapCHECK or MapCHECK 2) and back‐projects these errors into the patient's original treatment plan to perturb the original 3D patient doses. Note that the conventional γ analysis is not employed in this algorithm. Overlaying the calculated plane over measurement plane (using field‐by‐field (FBF) comparison technique) generates a 2D “error mask” plane (absolute dose differences) for each beam. Using the radiotherapy plan, structures, and dose imported from the TPS in DICOM format, 3DVH calculates dose contribution from each individual IMRT beam for each dose grid in patient based on ray tracing from the source to the dose grid. The dose of each voxel is perturbed for each beam using the beam's associated error mask. The 3DVH system modifies the error mask based on the depth inside the patient and distance from the source. Finally, the PDP error mask is summed for all voxels and all beams, generating a predicted dose distribution inside the patient. A more thorough evaluation of how PDP works has been discussed in Zhen et al.^(12)^ There are also other systems available such as the Compass system from IBA (Louvain‐la‐Neuve, Belgium), the ScandiDos (Uppsala, Sweden) Delta^4^ system, and Dosimetry Check software by Math Resolution (Columbia, MD) which can also perform DVH‐based QA. It should be noted that this type of QA does not work for volumetric‐modulated arc therapy (VMAT) since the fields can only be delivered perpendicular to the surface of the measuring device for 3DVH input. An SNC ArcCHECK PDP algorithm provides a solution to generate a perturbed dose distribution from a VMAT delivery and it is out of scope of this research.

The PDP algorithm requires a full density planar dose input. Because of the low detector density (diode spacing of 1.0 cm horizontally and 0.7 cm diagonally) of the MapCHECK 2 device, a method called “smarterpolation” was developed to generate a full density array from the MapCHECK 2 device, as explained in the SNC white paper.^(13)^ The smarterpolation is not a simple interpolation because it uses prior knowledge of dose gradients from the TPS to accurately increase the dose density. A study in the SNC white paper validates the accuracy of the smarterpolation algorithm by taking full density dose planes and sampling down the number of points to match a MapCHECK 2 density. The sampled down plane is inserted into the 3DVH system where it is converted back to a full density plane that is nearly equivalent to the original plane. When comparing the original full density and smarterpolated full density planes using γ test, 99.1% of points passed the agreement test using 2%/2 mm criteria.

A number of studies have shown the validity of 3DVH.^9^, ^10^, ^12^, ^14^, ^15^ Zhen et al.^(12)^ used 24 error‐free IMRT plans and introduced four types of errors to create 96 plans with errors. A correlation between the percent actual deviations (percent dose differences in DVH between error‐induced and error‐free plans) and the percent predicted deviations (percent dose differences in DVH between PDP‐predicted plan and error‐free plans) was investigated for each region of interest (ROI). For all ROIs, there was a strong correlation (for example, the CTV D95 had an $R^{2}$ value of 0.98534 with an ideal case being 1), proving that the PDP algorithm could accurately predict DVH‐based errors found in QA results of the error‐induced plans. On the other hand, Stasi et al.^(11)^ showed that there were weak correlations between clinically relevant dose differences in DVH reconstructed by 3DVH and GPRs. For instance, false negatives were found where high GPRs had high dose errors for certain ROIs. Because of the GPRs’ inability to predict patient dose errors, a transition to DVH‐based metrics is proposed to ensure proper treatment.

One of the main disadvantages of the SNC MapCHECK 2 is the low detector density, which can potentially affect the accuracy of the smarterpolation. Even though there have been an increasing number of studies for 3DVH, no study has yet been presented to determine the dependency of the PDP algorithm on the spatial resolution of the MapCHECK 2 detectors. The goal of this research has been to test how the detector density of MapCHECK 2 changes the output of the PDP calculation and, in addition, to investigate the dependency of 2D and 3D GPRs on spatial resolution and possible dose errors using 3DVH.

## II. MATERIALS AND METHODS

### A. IMRT verification plans

For this study, ten brain (small target), ten brain (large target; seven patients with total ten targets), ten prostate, and ten head‐and‐neck (H&N) IMRT verification plans were generated, as shown in Table 1. The clinical small brain plans were initially generated using the BrainLAB (Feldkirchen, Germany) iPlan TPS (version 4.5) and then exported to Varian (Palo Alto, CA) Eclipse TPS (version 8.9) for recalculation to have the same treatment planning environment. The large brain, prostate, and H&N plans were generated using the Varian Eclipse TPS with step‐and‐shoot technique. All plans were calculated using the anisotropic analytical algorithm (AAA) and a grid size of 2.0mm×2.0mm×2.0mm. Most of the H&N plans have multiple targets using simultaneous integrated boost. These treatment sites were chosen because of their PTV (planning target volume) size and different modulation complexity. The average PTV sizes (±a standard deviation(SD)) were 11.0±10.1cc (small brain), 293.4±165.6cc (large brain), 121.0±38.3cc (prostate), and 447.4±142.6cc (H&N), respectively. The degree of modulation complexity was estimated using total fractional MU divided by a fractional prescription dose (180 to 600 cGy) to the targets (MU/cGy). Even if this value is not an accurate measure of the complexity, the visual inspection showed it was a reasonable estimate. The order of increasing complexity was prostate (2.6±0.7MU/cGy), large brain (2.7±1.2MU/cGy), H&N (3.6±1.2MU/cGy), and small brain (3.9±0.7MU/cGy); however, it considerably varies even within the same group (Table 1).

**Table 1:** Summary of 40 IMRT QA plans studied along with their prescription dose and PTV size

	*Brain – Small Target*					*Brain – Large Target*				
*Patient Number*	*Treatment Site*	*# of Beams*	*PTV (cc)*	*Modulation Complexity (MU/cGy)*	*Energy*	*Treatment Site*	*# of Beams*	*PTV (cc)*	*Modulation Complexity (MU/cGy)*	*Energy*
1	Pituitary adenoma	11	21 .1	5. 3	6 MV	Astrocytoma (left)	8	330.1	1.4	6 MV
2	Cavernous sinus	9	7. 0	3. 6	6 MV	[Patient #1 boost]	5	195.9	1.4	6 MV
3	Metastatic (tonsil)	10	19 .1	3. 5	6 MV	Metastatic (right frontal)	5	59.0	2.3	6 MV
4	Meningioma (optic nerve)	10	17.7	4.6	6 MV	Brainstem	6	134.8	2.2	6 MV
5	Pituitary adenoma	10	6. 6	3. 8	6 MV	Glioblastoma (frontal)	7	547.7	2.0	6 MV
6	Right acoustic neuroma	10	1. 0	3. 6	6 MV	Glioblastoma (left frontal)	7	420.8	2.8	6 MV
7	Metastatic (cerebellar)	10	29 .0	4. 3	6 MV	(Patient #6 boost)	7	393.9	5.1	6 MV
8	Glomus paraganglioma	9	5. 1	2. 9	6 MV	Meningioma (left)	5	95.0	2.3	6 MV
9	Cerebral meningioma	10	2. 0	3. 8	6 MV	Glioblastoma (left)	8	453.1	2.5	6 MV
10	Right acoustic neuroma	10	0. 4	3. 7	6 MV	(Patient #9 boost)	8	303.6	4.7	6 MV
	*Prostate*					*Head and Neck* ^a^				
1	Prostate	7	156.3	2.7	10 MV	Nasopharynx	12	524.1	5.1	6 MV
2	Prostate	7	102.0	2.2	10 MV	Pharynx	12	526.7	4.2	6 MV
3	Prostate	9	131.1	2.6	6 MV	Right Parotid	5	288.2	1.6	6 MV
4	Prostate	9	100.1	1.8	10 MV	Right Parotid	9	309.8	2.2	6 MV
5	Prostate	9	80.1	3.1	10 MV	Esophagus	7	370.0	2.3	6 MV
6	Prostate	9	68.9	2.0	10 MV	Tongue	18	678.3	4.8	6 MV
7	Prostate	7	176.6	3.2	6 MV	Larynx	12	633.0	4.1	6 MV
8	Prostate	8	151.4	2.4	6 MV	Larynx	12	317.8	4.2	6 MV
9	Prostate	7	192.2	3.9	6 MV	Tonsil	14	495.1	3.8	6 MV
10	Prostate	7	51.4	2.0	6 MV	Tonsil	10	330.7	3.9	6 MV

a ^a^ Split beams were counted individually.

### B. IMRT QA delivery and devices

**Figure 1:**
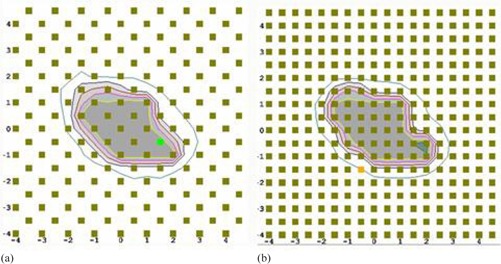
An example of the MapCHEK 2 QA measurements: (a) standard resolution (SR) measurement, and (b) high resolution (HR) measurement achieved by merging a shifted and a nonshifted measurement for MapCHECK 2.

All IMRT verification plans were delivered using the Varian TrueBeam STx with high definition MLC (HD120; leaf width of 2.5 mm in the center region (32 leaf pairs) and 5.0 mm in the outer part (28 leaf pairs)). The SNC MapCHECK 2 (serial number: 6959303) with MapPHAN‐MC2 was used to measure the dose distributions for all 40 IMRT validation plans. The MapCHECK 2 is a 2D array of 1527 n‐type diodes (an active area of 32.0cm(length)×26.0cm(width)) and the MapPHAN‐MC2 is a solid water block (34.9cm×37.9cm×8.0cm) with buildup of 5.0 cm water equivalent above and below the detector plane of MapCHECK 2. The MapCHECK 2 with MapPHAN‐MC2 was scanned using a GE CT scanner (GE Healthcare, Waukesha, WI) and transferred to the Eclipse TPS for dose calculations of IMRT QA plans on the phantom. The internal components of the MapCHECK 2 device produce significant CT artifacts which are especially pronounced on the lateral sides of the phantom scan. In this study, the raw CT dataset was used because all beams were delivered orthogonal to the front surface of the MapCHECK 2 where the effect of CT artifacts is limited. In addition, it was used to avoid any dosimetric uncertainties of overriding unknown HU values. The raw CT dataset produced a dose error of 0.6%±1.0% (13 diode points in the central region) for a $10\times 10\;{\text{cm}}^{2}$ square field measurement compared to the TPS calculation in the delivery setup. Measurements were performed for all 40 IMRT QA plans under two circumstances: measurement with normal detector density (SR; standard resolution) and measurement with the doubled detector density (HR; high resolution). Doubling the detector density is accomplished by delivering an IMRT QA plan on the MapCHECK 2 with normal alignment merged with another measurement of the same IMRT QA plan, but with the MapCHECK 2 manually shifted 5.0 mm right (patient right in supine, head‐first position), as shown in Fig. 1. The shift was measured by a ruler to achieve submillimeter accuracy. 3DVH provides a function of autoregistering QA pairs to find ideal matching (the best registration assuming no setup offset) between smarterpolated measurements and corresponding calculations. The effect of setup uncertainty was quantified by comparing data analyses without autoregistration (a 3D γ test and DVH analysis) to those with autoregistration on a per‐beam basis for all 40 targets using both SR and HR measurements. The X (MapCHECK 2 lateral) and Y (MapCHECK 2 longitudinal) offsets were separately detected. All measurements showed −1.0 to 1.0 mm setup error in either X or Y direction for both SR and HR QAs, except for one large brain case (patient #6: X=0.5mm and Y=−1.5mm). All other analyses were performed with the autoregistration off.

The MapCHECK 2 is normally calibrated with a 10cm×10cm field and the diode response is lower for small fields due to lack of scattered radiation (underdosing of about 1% for 6 MV).^(16)^ The standard 10cm×10cm field was used for calibration of the MapCHECK 2 device for the large brain, prostate, and H&N plans. However, for the small brain plans, a 2cm×3cm calibration field size was used in order to limit the dosimetric uncertainty.

### C. Dependency of PDP on the spatial resolution

Using SNC Patient software (version 6.0), error masks (SNCPDP files) were generated for the original (SR) and merged (HR) measurements (errors between the IMRT QA measurements and the 2D dose maps calculated by the TPS). These SNCPDP files were then fed into the SNC 3DVH system (version 1.1) along with DICOM CT images, RT dose, RT structures, and RT plan, which were imported from the Eclipse TPS. Using these data, 3DVH generated a new perturbed 3D dose distribution, DVH, and ROI dose statistics. Changes of dose coverage for PTV and organs at risk (OARs) were evaluated using D95 (dose that ≥95% of PTV receives), $D_{\text{mean}}$ (mean dose), and $D_{\text{max}}$ (maximum dose). For the small and large brain patients, $D_{\text{mean}}$ and $D_{\text{max}}$ of lens, eye, optic nerve, optic track (small brain only), optic chiasm, brainstem, and spinal cord (large brain only) were evaluated; while for the prostate patients, $D_{\text{mean}}$ and $D_{\text{max}}$ of bladder and rectum were evaluated. For the H&N patients, $D_{\text{mean}}$ and $D_{\text{max}}$ of lens, eye, cochlea, brainstem, submandible node, larynx, thyroid, mandible, spinal cord, and parotid gland were evaluated. Dose differences in DVH normalized to the prescription doses (Rx) were calculated for ROI dose statistics: $\Delta D_{\text{SR}}=(\text{perturbed dose with SR}-\text{planned dose})/\text{Rx}\times 100(\%),\Delta D_{\text{HR}}=(\text{perturbed dose with HR}-\text{planned dose})/\text{Rx}\times 100(\%)$, and $\Delta D_{\text{SR}-\text{HR}}=(\text{perturbed dose with SR}-\text{perturbed dose with HR})/\text{Rx}\times 100(\%)$. $\Delta D_{\text{SR}}$ was compared to $\Delta D_{\text{HR}}$ using the two‐tailed Student's t‐test for each ROI dose statistic. A p‐value less than 0.05 indicated statistically significant differences at the 95% confidence level.

### D. Correlation of GPR to PTV dose metrics in DVH

A 2D γ test (conventional planar IMRT QA) using the SNC patient software with nonsmarterpolated measurement points was performed for both SR and HR measurements. The average GPR for each patient was computed by applying a weighting factor proportional to MUs for each field. The 3D dose distribution from the original treatment planning was also compared to that of PDP calculation by the SR or HR measurement using the γ index in the SNC 3DVH software (global comparison: whole 3D distribution was compared). For both 2D and 3D γ tests, absolute comparisons with 10% threshold and three different criteria of 1%/1 mm (C1), 2%/2 mm (C2), and 3%/3 mm (C3) were employed. The statistical difference in 2D or 3D GPRs between SR and HR measurements was compared using the t‐test. In addition, correlations of the absolute change ($\left|\Delta D_{\text{SR}}\right|$ and $\left|\Delta D_{\text{HR}}\right|$) of PTV dose metrics ($D95,D_{\text{mean}}$, and $D_{\text{max}}$) to 2D or 3D GPRs were investigated for each measurement resolution (SR and HR) using Pearson product moment correlation values (r‐values) as described in Nelms et al.^(9)^ To quantify the MapCHECK 2 setup uncertainty, the correlations were also obtained with the 3DVH autoregistration on.

## III. RESULTS

### A. Dependency of PDP on the spatial resolution

The dose differences in $\text{DVH}\;(\Delta D_{\text{SR}},\Delta D_{\text{HR}}$, and $\Delta D_{\text{SR}-\text{HR}}$) are summarized in Tables 2 (brain, small target), 3 (brain, large target), 4 (prostate), and 5 (H&N). For the small brain plans, clinically substantial changes in the DVH metrics of PTV were observed for plans which have the smallest PTV sizes (patient #6 (1.0 cc): $\Delta D_{\text{SR}}=12.7\%$ and patient #10 (0.4 cc): $\Delta D_{\text{HR}}=11.0\%$ for $D_{\text{max}}$). Seven out of ten patients showed greater than 4% change of PTV $D_{\text{max}}$ in either $\Delta D_{\text{SR}}$ or $\Delta D_{\text{HR}}$. The mean absolute differences between $\Delta D_{\text{SR}}$ and $\Delta D_{\text{HR}}(|\Delta D_{\text{SR}-\text{HR}}|)$ of PTV D95 and $D_{\text{mean}}$ were larger than the other patients groups, and statistically significant difference was observed between the SR and HR measurements for D95 and $D_{\text{mean}}$. However, there was no statistically significant difference between SR‐predicted DVH and HR‐predicted DVH for OARs, as shown in the last column of Table 2 (p‐value ≥0.05) and the mean absolute differences ($\left|\Delta D_{\text{SR}-\text{HR}}\right|$) were less than 0.3%.

For large brain targets (Table 3), the changes in the DVH metrics of $\text{PTV}(D95,D_{\text{mean}}$, and $D_{\text{max}})$ were all less than 3% for both $\Delta D_{\text{SR}}$ and $\Delta D_{\text{HR}}$, which did not show any clinically meaningful impact in the 3DVH analysis evaluated by a physician. The mean differences in PTV coverage between the SR‐predicted DVH and the HR‐predicted DVH were not notably high (−1.2%±0.5% for D95 and $D_{\text{mean}}$ and −1.7%±0.5% for $D_{\text{max}}$); however, the difference was statistically significant. For all OARs, the mean $\left|\Delta D_{\text{SR}-\text{HR}}\right|$ was less than 1% (except for $D_{\text{max}}$ of brainstem (−1.1%±0.8%)); however, the statistical significance varied among the structures. In general, if a structure was proximal to PTV, the statistically significant difference between

**Table 2:** Percent dose differences for brain ‐ small target dose statistics, and comparison between standard and high resolution

			$\Delta D_{\text{SR}}(\%)$		$\Delta D_{\text{HR}}(\%)$		$\Delta D_{\text{SR}-\text{HR}}(\%)$		*p‐value*
*ROI (number of patients)*			*Mean (SD)*	*Range*	*Mean (SD)*	*Range*	*Mean (SD)*	*Range*	($\Delta D_{\text{SR}}$ *vs.* $\Delta D_{\text{HR}}$)
PTV (10)		D95	−0.2 (1.7)	[−3.9,1.0]	0.9 (1.3)	[−1.5,2.3]	−2.1 (0.9)	[−4.1,−1.3]	<0.0001
		$D_{\text{mean}}$	0.2 (1.0)	[−1.4,2.2]	1.9 (1.8)	[0.0, 5.8]	−1.8 (1.3)	[−5.2,−1.0]	0.002
		$D_{\text{max}}$	4.6 (3.9)	[−0.1,12.7]	5.0 (3.2)	[1.8, 11.0]	−0.4 (1.9)	[−2.8,3.5]	0.50
Lens (8)	Left	$D_{\text{mean}}$	−0.2(0.5)	[−1.4,0.2]	−0.1(0.5)	[−1.5,0.2]	0.0 (0.1)	[−0.3,0.1]	0.29
		$D_{\text{max}}$	−0.1(0.5)	[−1.2,0.3]	0.0 (0.5)	[−1.4,0.3]	−0.1 (0.2)	[−0.6,0.2]	0.39
	Right	$D_{\text{mean}}$	−0.2 (0.7)	[−2.2,0.2]	−0.1 (0.4)	[−1.0,0.3]	−0.1 (0.4)	[−1.2,0.1]	0.33
		$D_{\text{max}}$	−0.2 (0.7)	[−2.0,0.3]	0.1 (0.2)	[−0.3,0.5]	−0.3 (0.8)	[−2.3,0.1]	0.33
Eye (8)	Left	$D_{\text{mean}}$	−0.1 (0.3)	[−0.6,0.3]	−0.1 (0.2)	[−0.4,0.3]	0.0 (0.0)	[−0.1,0.0]	0.14
		$D_{\text{max}}$	−0.1 (1.2)	[−1.7,2.5]	0.0 (1.5)	[−2.3,2.3]	−0.1 (0.8)	[−2.0,0.6]	0.68
	Right	$D_{\text{mean}}$	−0.3 (0.6)	[−1.5,0.2]	−0.2 (0.6)	[−1.2,0.3]	0.0 (0.1)	[−0.2,0.0]	0.24
		$D_{\text{max}}$	−0.7 (1.9)	[−5.6,0.5]	0.4 (1.8)	[−2.9,3.2]	−1.1 (1.8)	[−4.0,0.2]	0.09
Optic nerve (8)	Left	$D_{\text{mean}}$	−0.0 (1.0)	[−1.3,2.3]	0.3 (0.9)	[−0.5,2.7]	−0.3 (0.4)	[−1.3,0.0]	0.07
		$D_{\text{max}}$	0.5 (1.3)	[−0.5,3.8]	0.7 (1.4)	[−0.4,4.0]	−0.1 (0.8)	[−1.8,1.1]	0.62
	Right	$D_{\text{mean}}$	−0.1(0.5)	[−0.9,0.8]	0.2 (0.4)	[−0.4,0.8]	−0.3 (0.4)	[−1.0,0.0]	0.08
		$D_{\text{max}}$	0.7 (1.4)	[−1.4,3.4]	1.1 (1.1)	[−0.1,2.6]	−0.4 (1.0)	[‐19, 1.2]	0.27
Optic track (6)	Left	$D_{\text{mean}}$	−0.8 (1.9)	[−5.0,0.3]	−0.6 (1.3)	[−3.3,0.3]	−0.2 (0.7)	[−1.7,0.6]	0.46
		$D_{\text{max}}$	−1.0 (2.4)	[−5.8,1.6]	−1.1 (1.9)	[−4.5,0.6]	0.1 (0.8)	[−1.3,1.0]	0.74
	Right	$D_{\text{mean}}$	0.1 (0.8)	[−0.9,1.5]	0.1 (1.1)	[−1.5,2.2]	0.0 (0.4)	[−0.7,0.7]	0.98
		$D_{\text{max}}$	−0.1 (2.0)	[−4.2,2.5]	0.1 (2.2)	[−3.6,4.0]	0.2 (0.7)	[−1.5,0.3]	0.46
Optic chiasm (8)		$D_{\text{mean}}$	0.1 (1.3)	[−2.8,2.0]	0.3 (1.7)	[−2.8,2.7]	−0.2 (0.6)	[−1.6,0.4]	0.37
		$D_{\text{max}}$	0.0 (1.4)	[−3.1,1.4]	0.1 (1.9)	[−3.3,2.9]	−0.1 (1.0)	[−1.7,1.8]	0.70
Brainstem (10)		$D_{\text{mean}}$	0.0 (0.3)	[−0.3,0.6]	0.1 (0.3)	[−0.2,0.7]	−0.1 (0.1)	[−0.2,0.1]	0.05
		$D_{\text{max}}$	−0.7 (1.6)	[−4.8,0.6]	−0.4 (1.7)	[−4.5,1.4]	−0.3 (0.7)	[−1.3,0.7]	0.20

$\Delta D_{\text{SR}}$ and $\Delta D_{\text{HR}}$ was observed (e,g., optic chiasm and brainstem; <3.0cm from the targets in most cases), whereas if the structure was away from the target (e.g., lenses and spinal cord), there was no statistically significant difference.

For the prostate plans (Table 4), in all of the compared PTVs and OARs (bladder and rectum) the difference between $\Delta D_{\text{SR}}$ and $\Delta D_{\text{HR}}$ was not clinically substantial ($\text{mean}|\Delta D_{\text{SR}}|,|\Delta D_{\text{HR}}|$, and $|\Delta D_{\text{SR}-\text{HR}}|\leq 1\%$), even if statistically significant differences were observed for D95 and $D_{\text{mean}}$ of PTV and bladder. For the H&N cases (Table 5), the differences between $\Delta D_{\text{SR}}$ and $\Delta D_{\text{HR}}$ were not also clinically substantial for all PTVs ($\text{mean}|\Delta D_{\text{SR}-\text{HR}}|\leq 1\%$). However, the differences were statistically significant for D95 and $D_{\text{mean}}$ of PTVs of higher prescription dose. The differences were negligible for all OARs ($\text{mean}|\Delta D_{\text{SR}-\text{HR}}|\leq 0.4\%$). However, if a structure is relatively proximal to the PTVs (e.g., larynx, thyroid, mandible, spinal cord, and parotid glands), the difference was statistically significant.

**Figure 2:**
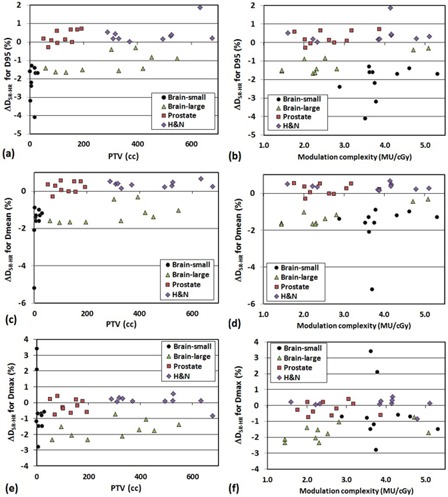
Difference of PTV dose statistics between SR and HR measurement ($\Delta D_{\text{SR}-\text{HR}}=\Delta D_{\text{SR}}$ $-\Delta D_{\text{HR}}$) as function of PTV size ((a) D95, (c) $D_{\text{mean}}$, and (e) $D_{\text{max}}$)) and modulation complexity ((b) D95, (d) $D_{\text{mean}}$, and (f) $D_{\text{max}}$)).

Figure 2 shows diagrams of $\Delta D_{\text{SR}-\text{HR}}$ for $D95,D_{\text{mean}}$, and $D_{\text{max}}$ of PTV with respect to the PTV size ((a), (c), and (e)) and the modulation complexity ((b), (d), and (f)) for all 40 QA plans. There was notably high difference in the PTV coverage between SR‐predicted DVH and HR‐predicted DVH as the PTV size decreased. Excluding the ten small brain cases whose PTV sizes were extremely small, the modulation complexity did not considerably change the PTV coverage (less than±2% for most of the cases).

**Table 3:** Percent dose differences for brain – large target dose statistics, and comparison between standard and high resolution

			$\Delta D_{\text{SR}}(\%)$		$\Delta D_{\text{HR}}(\%)$		$\Delta D_{\text{SR}-\text{HR}}(\%)$		*p‐value*
*ROI (number of patients)*			*Mean (SD)*	*Range*	*Mean (SD)*	*Range*	*Mean (SD)*	*Range*	($\Delta D_{\text{SR}}$ *vs.* $\Delta D_{\text{HR}}$)
PTV (10)		D95	−2.1(0.5)	[−3.0,−1.3]	−0.9 (0.8)	[−2.7,0.2]	−1.2(0.5)	[−1.6,−0.3]	0.0001
		$D_{\text{mean}}$	−1.5(0.5)	[−2.3,−0.8]	−0.3 (0.6)	[−0.8,0.8]	−1.2(0.5)	[−1.7,−0.3]	<0.0001
		$D_{\text{max}}$	−0.7 (0.8)	[−2.5,0.1]	1.0 (0.7)	[−0.2,2.3]	−1.7(0.5)	[−2.3,−0.7]	<0.0001
Lens (10)	Left	$D_{\text{mean}}$	−0.1 (0.2)	[−0.4,0.2]	−0.1 (0.2)	[−0.4,0.2]	0.0 (0.1)	[−0.2,0.1]	0.22
		$D_{\text{max}}$	−0.1 (0.2)	[−0.5,0.2]	−0.1 (0.2)	[−0.5,0.4]	−0.1 (0.1)	[−0.2,0.1]	0.17
	Right	$D_{\text{mean}}$	−0.2 (0.4)	[−1.1,0.3]	−0.2(0.5)	[−1.1,0.4]	0.0 (0.1)	[−0.2,0.2]	0.86
		$D_{\text{max}}$	−0.4 (1.0)	[−3.2,0.2]	−0.3 (0.7)	[−1.9,0.3]	−0.1(0.5)	[−1.3,0.3]	0.44
Eye (10)	Left	$D_{\text{mean}}$	−0.5 (0.9)	[−3.0,0.3]	−0.5 (0.9)	[−2.8,0.2]	0.0 (0.5)	[−0.3,1.4]	0.86
		$D_{\text{max}}$	−1.3 (1.5)	[−4.1,0.7]	−0.7 (1.4)	[−3.8,0.7]	−0.6 (0.8)	[−2.3,0.2]	0.04
	Right	$D_{\text{mean}}$	−0.2 (0.7)	[−1.8,0.8]	−0.3 (0.7)	[−1.6,0.1]	0.1 (0.8)	[−0.3,2.4]	0.62
		$D_{\text{max}}$	−0.4 (1.5)	[−4.3,1.2]	0.0 (1.6)	[−3.9,2.6]	−0.4(0.5)	[−1.4,0.0]	0.02
Optic nerve (10)	Left	$D_{\text{mean}}$	−0.7 (1.7)	[−5.4,0.4]	−0.3 (1.5)	[−4.5,0.9]	−0.4 (0.4)	[−0.9,0.0]	0.01
		$D_{\text{max}}$	−0.7 (1.3)	[−2.6,1.2]	0.0 (0.9)	[−1.3,1.3]	−0.7(0.5)	[−1.3,0.0]	0.002
	Right	$D_{\text{mean}}$	−0.6 (1.5)	[−4.4,0.8]	−0.2 (1.3)	[−3.7,0.9]	−0.3 (0.3)	[−1.0,0.0]	0.01
		$D_{\text{max}}$	−0.8 (1.5)	[−4.4,1.2]	0.0 (1.1)	[−2.8,1.3]	−0.8 (0.7)	[−2.2,0.0]	0.01
Spinal cord (10)		$D_{\text{mean}}$	−0.4 (0.7)	[−2.2,0.0]	−0.3(0.5)	[−1.6,0.0]	−0.1 (0.2)	[−0.5,0.0]	0.10
		$D_{\text{max}}$	−0.8 (1.0)	[−2.6,0.0]	−0.4 (0.7)	[−2.0,0.3]	−0.4 (0.9)	[−3.0,0.0]	0.20
Optic chiasm (10)		$D_{\text{mean}}$	−1.4 (1.8)	[−5.3,0.6]	−0.8 (1.6)	[−4.7,0.9]	−0.6(0.5)	[−1.3,0.0]	0.003
		$D_{\text{max}}$	−0.7 (1.5)	[−3.3,1.2]	−0.1 (1.2)	[−2.6,1.3]	−0.6(0.5)	[−1.5,0.0]	0.003
Brainstem (10)		$D_{\text{mean}}$	−1.7 (1.2)	[−4.3,−0.1]	−1.0 (1.0)	[−3.4,0.0]	−0.6 (0.6)	[−1.6,0.0]	0.01
		$D_{\text{max}}$	−1.6 (0.9)	[−2.6,0.3]	−0.6 (0.8)	[−2.4,0.3]	−1.1 (0.8)	[−2.3,0.1]	0.002

**Table 4:** Percent dose differences for prostate ROI dose statistics, and comparison between standard and high resolution

		$\Delta D_{\text{SR}}(\%)$		$\Delta D_{\text{HR}}(\%)$		$\Delta D_{\text{SR}-\text{HR}}(\%)$		*p‐value*
*ROI (number of patients)*		*Mean (SD)*	*Range*	*Mean (SD)*	*Range*	*Mean (SD)*	*Range*	($\Delta D_{\text{SR}}$ *vs.* $\Delta D_{\text{HR}}$)
PTV (10)	D95	−0.7 (1.5)	[−2.7,1.1]	−0.9 (1.6)	[−3.1,1.2]	0.3 (0.4)	[−0.3,0.7]	0.04
	$D_{\text{mean}}$	−0.6 (1.3)	[−2.5,1.0]	−0.8 (1.5)	[−2.7,1.0]	0.2 (0.3)	[−0.3,0.6]	0.04
	$D_{\text{max}}$	−0.6 (1.4)	[−2.4,1.4]	−0.4 (1.7)	[−2.3,1.8]	−0.2 (0.4)	[−0.7,0.4]	0.19
Bladder (10)	$D_{\text{mean}}$	0.5 (0.7)	[−0.8,1.4]	1.3 (0.5)	[0.6, 2.4]	−0.7 (0.7)	[−2.1,0.0]	0.01
	$D_{\text{max}}$	−0.3 (1.3)	[−2.2,1.0]	−0.2 (1.3)	[−2.7,0.9]	−0.1(0.5)	[−0.6,0.6]	0.49
Rectum (10)	$D_{\text{mean}}$	−0.4 (0.7)	[−1.5,0.4]	−0.5 (0.9)	[−1.8,0.5]	0.1 (0.2)	[−0.2,0.3]	0.31
	$D_{\text{max}}$	−0.5 (1.4)	[−2.7,1.3]	−0.5 (1.5)	[−3.2,1.2]	0.0 (0.3)	[−0.3,0.5]	0.59

**Table 5:** Percent dose differences for H&N ROI dose statistics, and comparison between standard and high resolution

			$\Delta D_{\text{SR}}(\%)$		$\Delta D_{\text{HR}}(\%)$		$\Delta D_{SR-HR}\;(\%)$		*p‐value*
*ROI (number of patients)*			*Mean (SD)*	*Range*	*Mean (SD)*	*Range*	*Mean (SD)*	*Range*	($\Delta D_{\text{SR}}$ *vs.* $\Delta D_{\text{HR}}$)
PTV>70 Gy (8)		D95	0.0 (0.6)	[−0.6,1.4]	−0.3 (0.7)	[−0.9,1.2]	0.3 (0.1)	[0.2, 0.5]	0.004
		$D_{\text{mean}}$	0.4 (0.7)	[−0.2,1.8]	0.1 (0.8)	[−0.6,1.6]	0.3 (0.1)	[0.2, 0.5]	0.0002
		$D_{\text{max}}$	1.0 (1.0)	[−0.8,3.0]	0.8 (1.4)	[−1.1,3.8]	0.1 (0.4)	[−0.8,0.6]	0.50
PTV>60 Gy (9)		D95	0.0 (0.7)	[−1.0,1.0]	−1.2 (1.3)	[−4.1,0.3]	1.0 (0.9)	[0.3, 3.1]	0.01
$D_{\text{mean}}$	0.7 (0.5)	[0.0, 1.4]	0.2 (0.5)	[−0.6,0.8]	0.5 (0.2)	[0.3, 0.9]	0.0001
$D_{\text{max}}$	1.3 (0.9)	[0.4, 3.1]	1.1 (1.0)	[−0.1,2.9]	0.1 (0.4)	[−0.7,0.7]	0.30
PTV>50Gy (8)		D95	−0.3 (0.9)	[−1.2,1.0]	−1.0 (1.7)	[−4.4,0.9]	0.7 (1.5)	[−1.4,3.4]	0.23
$D_{\text{mean}}$	0.6 (0.6)	[−0.1,1.7]	0.3 (0.7)	[−0.5,1.5]	0.4 (0.3)	[−0.1,0.8]	0.01
$D_{\text{max}}$	1.2 (0.6)	[0.3, 2.4]	1.1 (0.7)	[−0.2,2.2]	0.1 (0.3)	[−0.4,0.5]	0.28
Lens (8)	Left	$D_{\text{mean}}$	0.3 (0.4)	[0.0, 1.2]	0.3 (0.4)	[0.0, 1.1]	0.0 (0.0)	[−0.1,0.1]	0.87
		$D_{\text{max}}$	0.3 (0.4)	[0.0, 1.3]	0.3 (0.4)	[0.0, 1.3]	0.0 (0.1)	[−0.1,0.2]	0.84
	Right	$D_{\text{mean}}$	0.4 (0.8)	[−0.1,2.5]	0.4 (0.9)	[0.0, 2.5]	0.0 (0.0)	[−0.1,0.0]	0.46
	$D_{\text{max}}$	0.5 (1.1)	[−0.2,3.2]	0.5 (1.0)	[−0.1,3.0]	0.0 (0.1)	[−0.1,0.2]	0.32
Eye (7)	Left	$D_{\text{mean}}$	0.4 (0.6)	[0.0, 1.6]	0.4 (0.6)	[0.0, 1.7]	0.0 (0.0)	[0.0, 0.0]	0.17
		$D_{\text{max}}$	0.6 (1.1)	[0.0, 3.1]	0.6 (1.1)	[0.0, 3.1]	0.0 (0.0)	[0.0, 0.0]	0.32
	Right	$D_{\text{mean}}$	0.5 (1.0)	[−0.1,2.8]	0.5 (1.0)	[−0.2,2.8]	−0.1 (0.2)	[−0.5,0.0]	0.37
	$D_{\text{max}}$	0.7 (1.4)	[−0.2,3.8]	0.7 (1.4)	[−0.2,3.8]	0.0 (0.0)	[−0.1,0.0]	0.35
Cochlea (7)	Left	$D_{\text{mean}}$	0.5 (1.0)	[−0.3,2.7]	0.5 (1.0)	[−0.4,2.7]	0.0 (0.0)	[0.0, 0.0]	0.40
		$D_{\text{max}}$	0.4 (1.0)	[−0.5,2.4]	0.4 (1.0)	[−0.5,2.4]	0.0 (0.1)	[−0.1,0.1]	0.51
	Right	$D_{\text{mean}}$	0.8 (1.6)	[−0.5,4.2]	0.8 (1.5)	[−0.3,4.1]	0.0 (0.1)	[−0.2,0.1]	0.47
	$D_{\text{max}}$	0.8 (1.5)	[−0.5,4.0]	0.7 (1.5)	[−0.9,3.9]	0.0 (0.2)	[−0.1,0.3]	0.57
Brainstem (8)		$D_{\text{mean}}$	0.4 (1.2)	[−0.3,3.4]	0.4 (1.2)	[−0.4,3.4]	0.0 (0.1)	[0.0, 0.2]	0.10
		$D_{\text{max}}$	0.0 (1.4)	[−2.8,1.6]	0.0 (1.2)	[−1.9,1.7]	0.0 (0.4)	[−0.9,0.6]	0.83
Sub‐mandible node (5)	Left	$D_{\text{mean}}$	0.3 (0.8)	[−0.6,1.2]	0.1 (1.0)	[−1.0,1.1]	0.3 (0.2)	[0.1, 0.7]	0.07
		$D_{\text{max}}$	1.4 (0.8)	[0.3, 2.3]	0.9 (0.8)	[−0.4,1.6]	0.4 (0.3)	[0.1, 0.7]	0.03
	Right	$D_{\text{mean}}$	−0.3 (1.0)	[−1.9,0.6]	−1.0 (2.2)	[−4.8,0.3]	0.7 (1.2)	[0.0, 2.9]	0.25
	$D_{\text{max}}$	1.3 (1.1)	[−0.1,2.8]	1.0 (1.1)	[−0.1,2.7]	0.3 (0.2)	[0.1, 0.6]	0.07
Larynx (5)		$D_{\text{mean}}$	−0.9 (1.2)	[−3.0,0.1]	−1.3 (1.5)	[−3.9,−0.3]	0.4 (0.5)	[0.0, 0.9]	0.10
	$D_{\text{max}}$	0.3 (1.2)	[−0.8,2.2]	0.2 (1.5)	[−1.1,2.5]	0.1 (0.4)	[−0.4,0.6]	0.57
Thyroid (6)		$D_{\text{mean}}$	−0.8 (0.7)	[−1.7,0.0]	−1.0 (0.6)	[−1.8,−0.4]	0.3 (0.3)	[0.0, 0.7]	0.03
	$D_{\text{max}}$	0.2 (0.7)	[−0.7,1.0]	−0.3 (0.7)	[−1.1,0.7]	0.4 (0.1)	[0.3, 0.6]	0.001
Mandible (7)		$D_{\text{mean}}$	0.5 (0.3)	[−0.1,0.9]	0.3 (0.3)	[−0.2,0.8]	0.2 (0.1)	[0.0, 0.4]	0.004
	$D_{\text{max}}$	1.6 (1.3)	[0.0, 3.8]	1.2 (1.4)	[0.0, 3.7]	0.3 (0.2)	[0.1, 0.6]	0.02
Spinal cord (10)		$D_{\text{mean}}$	−0.3 (0.3)	[−0.6,0.1]	−0.5 (0.3)	[−1.0,0.0]	0.2 (0.1)	[0.1, 0.4]	0.001
	$D_{\text{max}}$	0.5 (0.6)	[−0.6,1.6]	0.3 (0.6)	[−0.6,1.2]	0.2 (0.4)	[−0.4,1.0]	0.16
Parotid	Left (9)	$D_{\text{mean}}$	0.1 (0.5)	[−0.4,1.3]	−0.1(0.5)	[−0.6,1.0]	0.1 (0.1)	[0.0, 0.3]	0.003
		$D_{\text{max}}$	1.1 (1.5)	[−2.6,2.4]	0.7 (1.6)	[−3.5,1.8]	0.4 (0.5)	[−0.7,0.8]	0.04
	Right (8)	$D_{\text{mean}}$	0.4 (0.5)	[0.0, 1.1]	0.1 (0.4)	[−0.3,1.0]	0.2 (0.2)	[0.0, 0.5]	0.01
		$D_{\text{max}}$	1.5 (0.7)	[0.6, 2.8]	1.1 (0.7)	[0.4, 2.5]	0.3 (0.4)	[−0.2,1.0]	0.05

### B. GPRs and PTV dose metrics in DVH

The average GPRs of the 2D and 3D γ test were all greater than 95% using the C3 criteria, as summarized in Table 6, which is considered clinically acceptable for the 2D γ test,^4^, ^5^ and differences in the mean GPR between SR and HR were all less than 1.0%, except for the 2D GPR of the small brain cases (1.6%). For the small brain QAs, patients #5 and #6 did not achieve 95% of 2D GPR (93.1%~94.7%) and patient #10 was unable to obtain 90% of points passing (95.8% for SR vs. 82.8% for HR); however, the GPR differences between SR and HR were statistically insignificant for all the criteria. The average 3D GPRs were significantly higher than the 2D GPRs for all criteria (p≪0.01).

For the large brain, prostate, and H&N cases, there was statistically insignificant difference between SR and HR for all 2D GPRs using C3, while statistically significant differences in 3D GPR were observed for prostate and H&N cases. Especially the difference was relatively large for the H&N cases (0.8%) compared to the other groups (0.3%~0.4%) whose modulation was more complex than the large brain and prostate cases. The difference between 2D GPR and 3D GPR was statistically insignificant for most cases (except for HR‐C1 of large brain (p=0.0001), SR‐C1 of H&N (p=0.008), and HR‐C3 (p=0.001) of H&N).

**Table 6:** 2D and 3D γ passing rates for three different γ criteria using standard and high resolution measurements

	*Cl* (1%/1 mm)				*C2* (2%/2 mm)				*C3* (3%/3 mm)			
	*2D*		*3D*		*2D*		*3D*		*2D*		*3D*	
	*SR*	*HR*	*SR*	*HR*	*SR*	*HR*	*SR*	*HR*	*SR*	*HR*	*SR*	*HR*
*Brain — Small Target* ($PTV_{size}=11.0\pm 10.1\;cc$)												
Mean	69.7	62.9	80.7	79.1	93.6	90.1	98.5	97.6	96.7	95.1	99.7	99.3
SD	6.3	8.0	9.7	8.3	3.5	6.2	1.3	2.3	1.9	4.8	0.4	0.7
*p‐value* (SR vs. HR)	0.06		0.08		0.10		0.07		0.25		0.05	
*Brain — Large Target* ($PTV_{size}=293.4\pm 165.6\;cc$												
Mean	70.1	70.7	68.3	79.1	95.8	95.4	94.4	96.6	99.6	99.5	98.9	99.2
SD	10.3	9.3	8.9	11.2	4.4	3.4	5.5	4.5	0.7	0.4	1.8	1.0
*p‐value* (SR vs. HR)	0.74		0.0003		0.66		0.01		0.59		0.26	
*Prostate* ($PTV_{size}=121.0\pm 38.3\;cc$)												
Mean	76.4	68.0	78.9	69.8	97.2	94.6	98.0	95.5	99.8	99.7	99.9	99.5
SD	6.7	9.3	4.4	9.0	1.9	2.7	1.0	1.5	0.3	0.4	0.2	0.5
*p‐value* (SR vs. HR)	0.005		0.01		0.01		0.002		0.33		0.03	
*Head and Neck* ($PTV_{size}=447.4\pm 142.6\;cc$)												
Mean	78.1	79.5	80.8	81.1	96.3	96.8	97.3	96.7	99.4	99.6	99.5	98.7
SD	4.5	3.3	5.8	5.4	2.0	1.5	1.5	1.6	0.6	0.4	0.6	0.8
*p‐value* (SR vs. HR)	0.03		0.57		0.04		0.01		0.11		0.0002	

**Figure 3:**
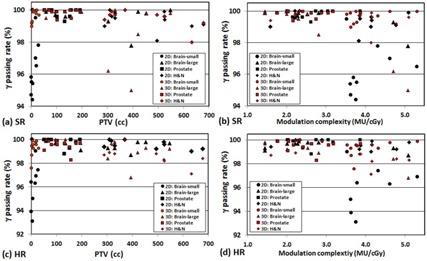
2D and 3D γ passing rates using 3%/3 mm criteria for SR and HR measurement with respect to PTV size and modulation complexity. One extreme outlier (82.8% for 2D HR for the brain – small target patient #10) was excluded from panels (c) and (d) to better show spreads of γ passing rates.

Figure 3 illustrates relationship of the GPR at the C3 criteria with the PTV size and the modulation complexity. A remarkable drop of GPR was shown as the PTV size decreased for both the (a) SR and the (c) HR QA measurements. It also shows several drops in GPRs at larger PTVs. It can be explained with the fact that plans with more complex modulation tend to have lower GPR, as shown in Figs. (b) and (d). Especially, the 3D GPR was lower than 2D GPR for some plans with relatively complex modulation and large targets for both SR and HR QAs (triangle and diamond markers). A similar relationship between low GPR and high plan modulation was observed at the C2 level; however, it diminished at the C1 level due to a wide spread of 2D and 3D GPRs.

**Figure 4:**
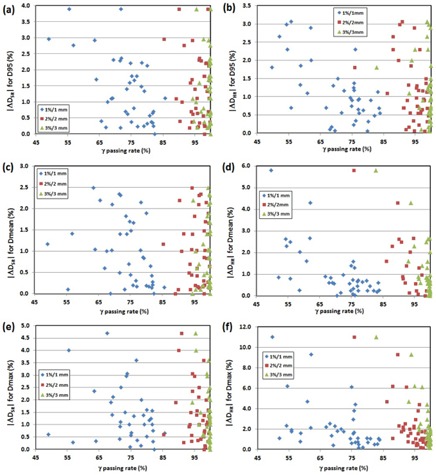
Scatter diagram between dose difference (original plan vs. PDP‐perturbed plan) and 2D γ passing rates for PTV dose statistics: (a) $\left|\Delta D_{\text{SR}}\right|$ and (b) $\left|\Delta D_{\text{HR}}\right|$ for D95, (c) $\Delta D_{\text{SR}}|$ and (d) $\left|\Delta D_{\text{HR}}\right|$ for $D_{\text{mean}}$, and (e) $\left|\Delta D_{\text{SR}}\right|$ and (f) $\left|\Delta D_{\text{HR}}\right|$ for $D_{\text{max}}$.

**Figure 5:**
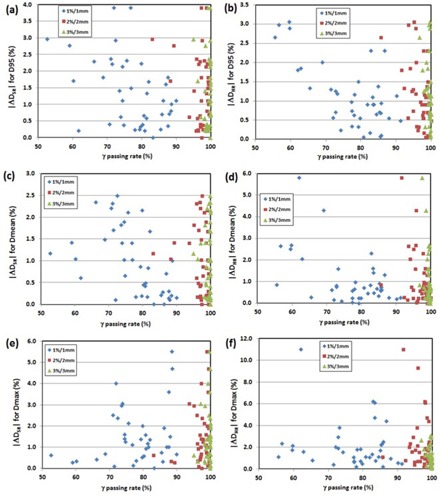
Scatter diagram between dose difference (original plan vs. PDP‐perturbed plan) and 3D γ passing rates for PTV dose statistics: (a) $\left|\Delta D_{\text{SR}}\right|$ and (b) $\left|\Delta D_{\text{HR}}\right|$ for D95, (c) $\left|\Delta D_{\text{SR}}\right|$ and (d) $\left|\Delta D_{\text{HR}}\right|$ for $D_{\text{mean}}$, and (e) $\left|\Delta D_{\text{SR}}\right|$ and (f) $\left|\Delta D_{\text{HR}}\right|$ for $D_{\text{max}}$.

The scatter diagrams between GPR and difference in DVH metrics of PTV are shown in Fig. 4 (2D GPR) and Fig. 5 (3D GPR). There were moderate (0.3<|r|<0.7) to strong (0.7≤|r|) correlations between DVH‐based QA metrics and IMRT QA performance metrics with several exceptions, as shown in Table 7. The correlations tend to be stronger with the tighter tolerance for both 2D and 3D QA metrics. This is probably because the distribution of 2D and 3D GPRs becomes narrower as the QA tolerance is looser. In a majority of cases, stronger correlation was also observed for the HR measurement for both 2D and 3D. Interestingly, the Pearson r‐values were dominantly negative indicating that smaller clinical errors occurred at the higher GPRs for 2D and 3D.

**Table 7:** Pearson r‐values correlating γ passing rates (2D and 3D) to absolute percent dose difference ($\left|\Delta D_{\text{SR}}\right|$ or $\left|\Delta D_{\text{HR}}\right|$) for PTV ROIs: D95, Dmean, and $D_{\text{max}}$

	*DVH*	*C1*		*C2*		*C3*	
	*metrics*	(1%/1 mm)		(2%/2 mm)		(3%/3 mm)	
	*(PTV)*	*SR*	*HR*	*SR*	*HR*	*SR*	*HR*
2D gamma test	D95	−0.56	−0.64	−0.17	−0.40	0.04	−0.19
	$D_{\text{mean}}$	−0.31	−0.65	0.02	−0.76	0.15	−0.77
	$D_{\text{max}}$	−0.28	−0.48	−0.32	−0.73	−0.65	−0.84
2D gamma test(autoregistration)	D95	−0.49	−0.70	−0.16	−0.64	0.14	−0.46
	$D_{\text{mean}}$	−0.50	−0.72	−0.23	−0.85	0.08	−0.82
	$D_{\text{max}}$	−0.48	−0.55	−0.61	−0.80	−0.74	−0.91
3D gamma test	D95	−0.43	−0.66	−0.23	−0.48	−0.02	−0.36
	^D^mean	−0.51	−0.52	−0.16	−0.33	0.00	−0.37
	$D_{\text{max}}$	−0.11	−0.20	0.09	−0.16	0.02	−0.35
3D gamma test (autoregistration)	D95	−0.55	−0.64	−0.30	−0.54	−0.16	−0.33
	$D_{\text{mean}}$	−0.66	−0.62	−0.44	−0.61	−0.27	−0.46
	$D_{\text{max}}$	0.01	−0.34	−0.02	−0.44	−0.08	−0.39

### C. Effect of setup uncertainty of MapCHECK 2

The small brain cases were most susceptible to the setup errors. For instance, patient #6 (PTV=1.0 cc) and #10 (PTV=0.4 cc) showed 8.6% (4.1% (auto) and 12.7% (nonauto)) and 7.3% (2.5% (auto) and 9.8% (nonauto)) change of $\Delta D_{\text{SR}}$ in the PTV $D_{\text{max}}$ coverage, respectively, with X=0.5 mm and Y=−0.5 mm offset. Most of the small brain measurements had ±0.5 mm offset for both SR and HR, which produced average changes between autoregistration and nonautoregistration from $-0.6\%\pm 1.1\%(\Delta D_{\text{HR}}$ for brainstem) to $0.7\%\pm 0.9\%(\Delta D_{\text{SR}}$ for D95). In addition, significant improvement in 3D GPR was observed using the tighter criteria for both SR and HR (e.g., 87.7%±6.0% (auto) vs. 80.7%±9.7% (nonauto) for SR‐C1 and 84.4%±8.2% (auto) vs. 79.1%±8.3% (nonauto) for HR‐C1). However, there was no statistically significant difference in 3D GPR using the C3 criteria (99.9%±0.1% (auto) vs. 99.7%±0.4% (nonauto) for SR and 99.4%±1.1% (auto) vs. 99.3%±0.7% (nonauto) for HR).

For the large brain, prostate, and H&N cases, the similar offsets were detected (X=0.0 to 0.5 mm and Y=−1.5 to −0.5 mm). The maximum coverage change detected with X=0.0±0.0 mm and Y=−0.8±0.2 mm was −1.8%±0.8% in $D_{\text{mean}}$ of bladder (mean ($\Delta D_{\text{HR}}$(auto) ‐ $\Delta D_{\text{HR}}$(nonauto))) for the ten prostate plans. In most of cases the mean changes in $D_{\text{mean}}$ and $D_{\text{max}}$ were less than 0.5% with the setup error of ~1 mm. Statistically significant improvements in 3D GPR were observed for the C1 criteria after the autoregistration with plans whose setup errors were detected (21 plans for SR and 20 plans for HR out of 30 plans; e.g., 87.1%±6.9% (auto) vs. 74.9%±9.6% (nonauto) for SR‐C1 and 84.9%±7.3% (auto) vs. 74.9%±10.6% (nonauto) for HR‐C1); however, the change of 3D GPR with C3 was not substantial (99.3%±1.3% (nonauto) to 99.8%±0.5% (auto) for SR and 99.3%±0.9% (nonauto) to 99.7%±0.7% (auto) for HR). Finally, the autoregistration generally amplified the correlations between GPR and DVH metrics ($D95,D_{\text{mean}}$, and $D_{\text{max}}$ of PTV) as shown in Table 7 indicating the 2D/3D GPRs based on more accurate measurement setup will better show clinically relevant dose changes in DVH.

## IV. DISCUSSION

### A. 2D and 3D γ passing rates

**Figure 6:**
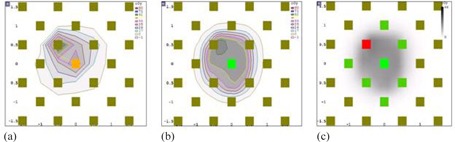
An example of 2D γ test for a small field (brain – small target patient #10): (a) measurement, (b) TPS calculation, and (c) γ test result; green=passed and red=failed (measurement higher). Total 7 out of 8 points passed the QA test (GPR=87.5%).

For the small brain QAs, the 3D GPRs were higher than the 2D GPRs (Table 6). These higher passing rates occur because the 3D γ test uses more points that can be searched and thus has a higher chance of achieving a γ value less than one (number of comparison points: $4.7\times 10^{4}\pm 2.6\times 10^{4}$ (3D SR and HR) vs. 30±17 (2D SR) vs. 60±30 (2D HR)). This study showed that three small brain plans were not able to achieve 95% of points passing the γ criteria (even 90%) using the 2D γ test at C3. This is due to limitation of the γ test when measuring small fields with low resolution detectors, such as MapCHECK 2. If there are a small number of points, such as the case with many of the small brain fields, only a small number of failed points are needed to drop the GPR below the common standard of 95%, as shown in Fig. 6 (patient #10).

For the other patient groups, the differences between 2D and 3D GPRs were not as noticeably high as the small brain cases. This is because the number comparison points of 2D γ test were three (prostate: 96±22 for SR) to seven (H&N: 220±44 for SR) times more than the small brain cases, and thus the chance of undersampling effect (Fig. 6) is much less. For some cases, the 3D GPR was lower than the 2D GPR at the C3 level. This occurs when nearly all the points pass on the 2D plane. They will still pass the 3D test when inserted into 3DVH because it does not change the value of measured points. However, when the low density MapCHECK 2 measurement is put in 3DVH, the smarterpolation algorithm converts it to a full density measurement where the measured plane has more points that may fail. The opposite situation, where a measurement with low 2D GPR comes up with a high 3D GPR, was also observed.

In most cases (30 (2D) and 32 plans (3D) out of 40 comparisons), the HR measurements had lower GPRs than the SR measurements at C3. This happens because the points that failed in the SR measurement tend to also fail in the HR measurement and, since there are more points in the HR measurements with shifted measurements, other points are likely to fail as well. Our results also showed no statistically significant difference between SR and HR measurements for all four types of plans in routine IMRT QA at the γ criteria of 3%/3 mm using 2D γ test. Merging of the MapCHECK 2 measurements is not necessary for these four treatment sites in the conventional planar IMRT QA, because the HR measurement will give an almost identical GPR as the SR measurement. However, this study showed a stronger correlation of GPR to dose errors when the HR measurement was used, indicating that it is a better indicator of clinical dose errors (Table 7). The HR measurement may be also beneficial in the 3D γ test especially for the more complex targets, such as H&N, since statistically significant difference between SR and HR was observed at C3 (Table 6). It should be noted that there are few studies on the acceptable GPR for the 3D γ test. In this study, statistically insignificant differences were found between 2D GPR and 3D GPR for most cases; however, further studies are necessary to set clinically acceptable 3D GPR with various dose difference/DTA criteria.

One more possible error source in the γ tests is calibration of MapCHECK 2. The standard 10cm×10cm field for calibration increases the dosimetric uncertainty of field sizes less than 5cm×5cm. Therefore, in the case of small fields the percentage of points passing the γ test should be carefully examined. Even if a calibration field size of 3cm×3cm was used for the small brain cases, beam segments were sometimes much smaller than 3cm×3cm for which an uncertainty of an ionization chamber measurement for dose calibration is too high to be accepted. For the large targets, the similar problem existed with the 10cm×10cm calibration where subfields or island fields in a beam segment were sometimes much smaller than the calibration field size. Further studies are needed to find an optimal calibration field size of MapCHECK 2 considering differential response of diode to different beam energies. Our preliminary study showed ~0.7% dosimetric difference between the two different calibrations sizes for 6 MV.

### B. Dose metrics changes in 3DVH by detector resolution

Our result showed comparable errors provided by Stasi et al.^(11)^ They showed average errors of −2.11%, −1.78%, and −0.69% for PTV boost D95 (prostate), PTV boost $D_{\text{mean}}$ (prostate), and bladder $D_{\text{mean}}$, respectively. For the same ROIs, our study showed −0.7%±1.5%, −0.6%±1.3%,0.5%±0.7% for SR, and −0.9%±1.6%, −0.8%±1.5%,1.3%±0.5% for HR, respectively. The purpose of this study was to investigate the effect of detector resolution on DVH‐based QA metrics in pretreatment dose QA. The differences between SR and HR for the PTV coverage were statistically significant for small brain, large brain, and H&N cases and relatively smaller for the prostate cases. This indicates that the HR measurement may be more beneficial to accurately determine the change in PTV coverage for more complex targets. In general, the difference in DVH metrics between SR and HR measurements was largest in the small brain QAs, and it was observed that the difference in PTV dose metrics dramatically increased as the PTV size decreased, as shown in Fig. 2. Excepting the small brain cases, the modulation complexity does not make any clinically considerable changes in PTV dose metrics. If the PTV size is greater than approximately 5 cc (e.g., brain, small target patient #8), in most cases there was no substantial difference between SR‐predicted and HR‐predicted DVH‐based QA metrics for all ROIs (most were less than 2%). This indicates that 3DVH may not make any clinically substantial difference between SR and HR QA measurements when the PTV size is greater than 5 cc. It begs the question, “Should the HR‐predicted DVH‐based QA metrics be used for PTV size <5 cc?” Answering this question is not an easy task because even the HR measurement has relatively low‐density resolution compared to other high‐density resolution dosimetry such as film or electronic portal imaging device (EPID), and accuracy of the MapCHECK measurement should also be considered. For instance, the brain ‐ small target patient #10 (PTV=0.4 cc) had only an average of 15 diode points for per‐beam comparison, even for the HR measurements. Furthermore, these comparison points were located on high gradient regions where a small displacement of 1 mm may cause dose errors in the range of 10%–20%. The autoregistration study showed that the setup error of 0.5 mm in both X and Y directions propagated to 7.3% change of $\Delta D_{\text{SR}}$ (PTV $D_{\text{max}}$) in 3DVH for patient #10. To achieve the best outcome of DVH‐based QA metrics for small targets (PTV<5 cc), three conditions should be met: (1) very high‐density resolution of 2D dosimeter (preferably equivalent to film dosimetry), (2) submillimeter accuracy of QA measurement setup, and (3) accurate calibration of the dosimeter. Considering all these prerequisites, it might not be highly recommended to use 3DVH for the DVH‐based QA for small targets. If it is inevitably used, the HR QA measurement should be performed because it significantly reduces the 3DVH prediction error with setup errors of ~1 mm (e.g., $\Delta D_{\text{SR}}(\text{auto}-\text{nonauto})=-7.3\%$ to $\Delta D_{\text{HR}}(\text{auto}-\text{nonauto})=-0.3\%$ for PTV $D_{\text{max}}$ of patient #10). Further studies to determine if using higher density measurements would change the output of 3DVH and determining suitable DVH‐based metrics for IMRT QA need to be performed.

This research is only valid if 3DVH can accurately predict patient dose errors based on errors found in IMRT QA. As stated before, there have been many studies to validate that 3DVH works^9^, ^10^, ^12^, ^14^, ^15^ and our study is based on accuracy of the PDP algorithm from these validation studies. Another issue with this study was the inability to increase the detector density to a higher amount. It is possible that doubling the detector density is not sufficient enough to see changes in DVH metrics or GPRs. Another intrinsic problem was that 3DVH does not take into account other errors such as inter‐/intrafractional motion of targets and organs.

### C. Correlation between DVH‐based QA metrics and GPR

In this study, moderate‐to‐strong correlations were observed between DVH‐based QA metrics of PTV ($D95,D_{\text{mean}}$, and $D_{\text{max}}$) and GPR (both 2D and 3D), as shown in Table 7. The correlations tend to be stronger for HR and autoregistration. It implies that a patient‐specific QA with higher resolution and less setup error has a better potential to accurately predict clinically relevant dose errors. The dominant negative Pearson r‐values for the correlation between DVH‐based QA metrics of PTV and GPR indicates that the plans with higher 2D or 3D GPRs more likely contain smaller clinical errors at least in the PTV. A similar result was also reported by Stasi et al.^(11)^

Contrary to previous studies,^9^, ^11^, ^12^ the correlation of the absolute change of OAR metrics to GPRs was not assessed in this study. The 10% threshold (diode dose points below 10% maximum dose are ignored) for the 2D or global 3D γ test sometimes excluded majority of dose points in the OARs from QA comparison (especially in the small brain QAs) which resulted in insufficient sampling of dose points for comparison. For this reason, it is to some extent obvious that there are weak correlations between DVH‐based QA metrics and GPR for OARs observed in the previous studies. One more basic limitation of this study was inconsistency in the sizes and locations of the targets and OARs for the patients. Relatively larger variation in DVH metrics of OARs for the small brain patients originated from different degree of proximity of the OARs to PTV (Table 2). In general, statistically significant differences in the DVH statistics between SR and HR were found when OARs were proximal to PTVs and the plan modulation was relatively complex. The HR measurement is thus recommended for plans with a number of critical structures abutting targets and relatively high modulation complexity such as H&N cases in 3DVH analysis.

Another limitation of this study was the IMRT QA delivery technique. All fields were delivered at a gantry angle of zero instead of the actual angles used in patient delivery. This technique eliminates possible errors found due to gantry sag and gravity effects on MLC leaf motion. The FBF delivery at the patient angles can be accomplished using an isocentric mounting fixture (IMF). The delivery using the IMF was not performed in this study because a safety lock of the device does not allow the MapCHECK 2 to be shifted which would have prevented us from obtaining higher resolution measurements, and setup offsets of |X|=2 mm and Y=1 mm were observed at gantry angles of 90° and 270° due to sagging by IMF itself. A study comparing ROI dose statistics obtained using 3DVH for these two techniques showed considerable differences between the techniques. That study stated that “per‐beam IMRT QA should be conducted at gantry angles as designed for the patient treatment in order to obtain true clinical dose metrics”.^(17)^ However, the study's authors did not consider differential migration of central axis of MapCHECK caused by sagging of IMF itself depending on gantry angles.

## V. CONCLUSIONS

Differences in 2D GPRs between SR and HR MapCHECK QA were found to be statistically insignificant at 3%/3 mm for all small target (brain), large target (brain), prostate, and H&N plans. When using the percentage of points passing the γ test as a QA criterion, the HR measurements may not be necessary for conventional 2D planar IMRT QA, except for the small brain cases. Moderate to strong correlation between GPRs (2D and 3D) and the PTV dose statistics in DVH was found. In nearly all cases, the Pearson r‐values were negative, indicating that small clinical errors occur at the higher GPRs for PTV dose metrics. This indicates that the γ test has a strong ability to detect clinically relevant dose errors in PTV. However, this result does not show that the γ test is sensitive enough to catch all errors. Our results show mean difference of less than 2.0% between $\Delta D_{\text{SR}}$ and $\Delta D_{\text{HR}}$ for almost all ROI dose statistics and plans, indicating that doubling the detector resolution of the MapCHECK 2 does not heavily affect the PDP algorithm in 3DVH. However, it was also found that dose differences in DVH statistics between standard and high resolution were statistically significant for OARs proximal to PTVs when smaller planning target volumes and highly modulated plans were used for 3DVH analysis. Thus it is recommended to use the high resolution measurement for small targets (i.e., PTV<5 cc) and multiple targets with complex geometry with minimum setup error.

## Supporting information

Supplementary Material

Supplementary Material

Supplementary Material
