# Supplementary figures and images for: Dependency of planned dose perturbation (PDP) on the spatial resolution of MapCHECK 2 detectors
Source: J Appl Clin Med Phys. 2014 Jan 6;15(1):100–17. doi: 10.1120/jacmp.v15i1.4457 (PMC5711226; doi:10.1120/jacmp.v15i1.4457)

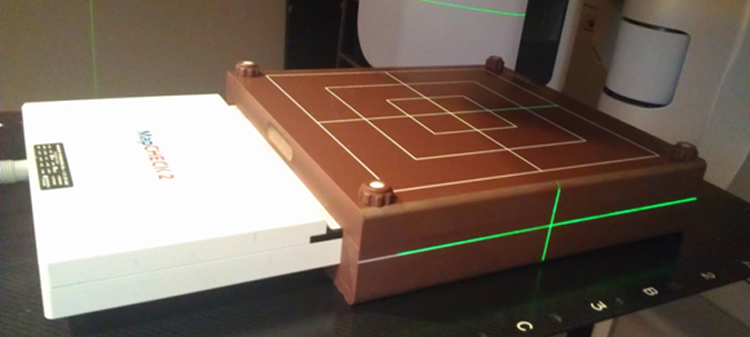

Supplement: Supplementary file 1 — Supplementary Material [file ACM2-15-100-s001.jpg]

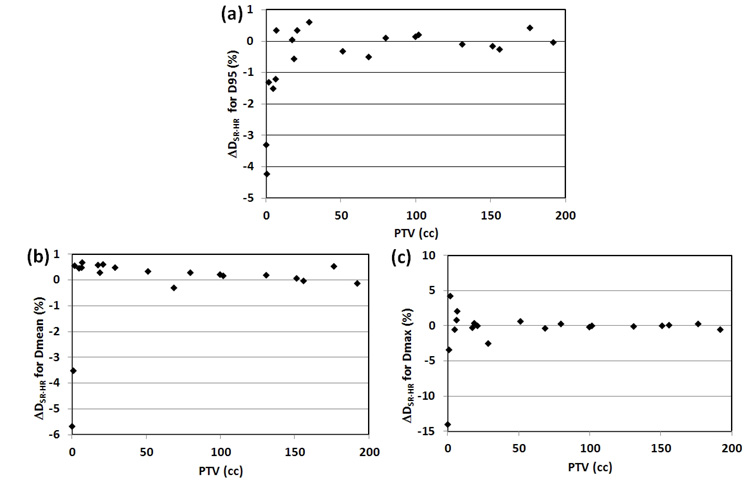

Supplement: Supplementary file 2 — Supplementary Material [file ACM2-15-100-s002.jpg]

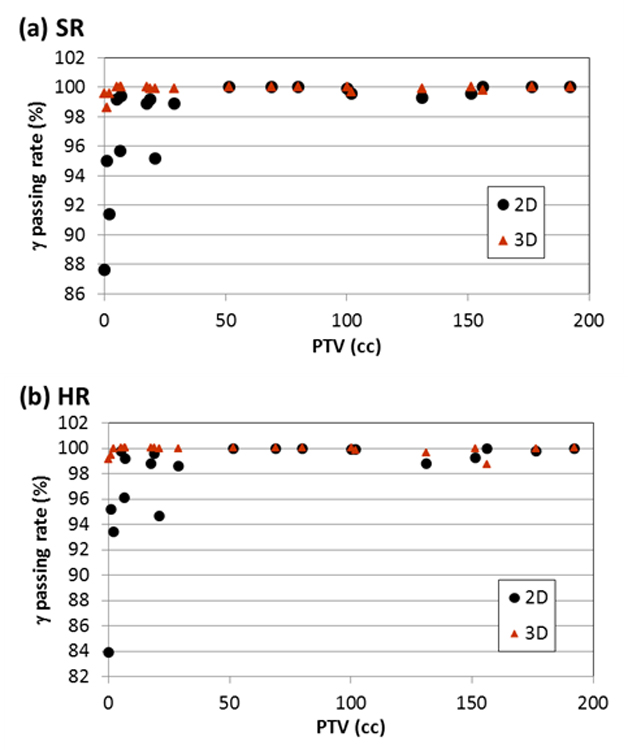

Supplement: Supplementary file 3 — Supplementary Material [file ACM2-15-100-s003.jpg]
